# Predicting short-term survival after liver transplantation on eight score systems: a national report from China Liver Transplant Registry

**DOI:** 10.1038/srep42253

**Published:** 2017-02-13

**Authors:** Qi Ling, Haojiang Dai, Runzhou Zhuang, Tian Shen, Weilin Wang, Xiao Xu, Shusen Zheng

**Affiliations:** 1 Division of Hepatobiliary and Pancreatic Surgery, Department of Surgery, Collaborative innovation center for diagnosis and treatment of infectious diseases, the First Affiliated Hospital, Zhejiang University School of Medicine, Hangzhou, China

## Abstract

To compare the performance of eight score systems (MELD, uMELD, MELD-Na. iMELD, UKELD, MELD-AS, CTP, and mCTP) in predicting the post-transplant mortality, we analyzed the data of 6,014 adult cirrhotic patients who underwent liver transplantation between January 2003 and December 2010 from the China Liver Transplant Registry database. In hepatitis B virus (HBV) group, MELD, uMELD and MELD-AS showed good predictive accuracies at 3-month mortality after liver transplantation; by comparison with other five models, MELD presented the best ability in predicting 3-month, 6-month and 1-year mortality, showing a significantly better predictive ability than UKELD and iMELD. In hepatitis C virus and Alcohol groups, the predictive ability did not differ significantly between MELD and other models. Patient survivals in different MELD categories were of statistically significant difference. Among patients with MELD score >35, a new prognostic model based on serum creatinine, need for hemodialysis and moderate ascites could identify the sickest one. In conclusion, MELD is superior to other score systems in predicting short-term post-transplant survival in patients with HBV-related liver disease. Among patients with MELD score >35, a new prognostic model can identify the sickest patients who should be excluded from waiting list to prevent wasteful transplantation.

The Model for End-stage Liver Disease (MELD) scoring system was developed to estimate the survival of patients undergoing transjugular intrahepatic portosystemic shunts^1^. It has been validated as a good predictor of mortality for a broader range of patients with end-stage liver disease, including candidates on the waiting list for liver transplantation^1^. Since 2002, MELD has been implemented as a liver allocation tool in the USA. It achieved great improvement in donor liver allocation compared with Child-Turcotte-Pugh (CTP)^2^ and is widely used for organ allocation nowadays. However, MELD has some limitations. Above all, cirrhotic complications such as persistent ascites and hyponatremia, which could contribute to poor prognosis, are not included in the MELD formula. Therefore, a series of modified MELD formulas have been developed to better predict the patient survival on the waiting list^3^,^4^,^5^,^6^,^7^,^8^. A study evaluated the prognostic ability of six prognostic formulas in 487 candidates with cirrhosis and found MELD-sodium (MELD-Na) and integrated MELD (iMELD) were better prognostic models than MELD to predict the drop-out rate among patients awaiting transplantation^9^. Although the predictive value of MELD and its modified formulas in liver transplant candidates has been well established, there are limited data focusing on the post-transplant survival.

In the present study, we aim to evaluate the efficacy of pre-transplant MELD and other scoring systems in the prediction of post-transplant survival using a large cohort of patients from the China Liver Transplant Registry (CLTR) database. With the most accurate prognostic prediction model recognized, the secondary objective is to identify patients who should be excluded from waiting list to prevent wasteful transplantation.

## Research Design and Methods

### Study population

The data used in this study was extracted from CLTR database. The study population included adult cirrhotic patients (>18 years) who underwent liver transplantation between January 2003 and December 2010 (n = 14,220). We excluded those who underwent re-transplantation (n = 449) or combined liver-kidney transplantation (n = 185), or had hepatocellular carcinoma (n = 7,146). Another 762 patients were not accounted because of incomplete laboratory and clinical data. The data from the remaining 6,014 patients were enrolled and used for analysis. Patient characteristics were showed in Table 1. All study patients were routinely followed up at outpatient clinic for at least 3 months, and the study on a patient concluded when he/she died. This study was approved by the Scientific Committee of CLTR (Approval No. 458; http://www.cltr.org/), which was authorized as the only national liver transplantation registry in Mainland China by the Ministry of Health. CLTR started the research after obtaining the approval of Ethics Committee from each participating centre (http://www.cltr.org/pages/trancenter/trancenter_map.jsp/) according to the Regulations on Human Organ Transplant and national legal requirements. The procedures were in accordance with the Helsinki declaration of 1975, as revised in 1983. All persons gave their informed consent prior to their inclusion in the study.

### Data collection

The following data were recorded: age, gender, blood groups, etiologies (hepatitis B virus [HBV], hepatitis C virus [HCV], alcohol and others), complications (ascites, hepatic encephalopathy, hepatorenal syndrome, gastrointestinal bleeding, and spontaneous peritonitis), comorbidities (diabetes mellitus, hypertension, and sepsis), serum biochemistry (albumin, bilirubin, creatinine, international normalized ratio, prothrombin time, and sodium), hemodialysis, and operation history. All laboratory data were the last recorded values before transplantation.

### Calculation of prognostic scores

The MELD score was calculated with the standard formula as 3.78 × ln (bilirubin [mg/dL]) + 9.57 × ln (creatinine [mg/dL]) + 11.20 × ln (international normalized ratio) + 6.43 × (0 if cholestatic or alcoholic, 1 otherwise)^1^. Minimal value for parameter of MELD score was 1 and maximal value for creatinine was 4. Creatinine was set at 4 if the patient was receiving renal replacement therapy.

The updated MELD (uMELD) score was calculated as 1.266 × ln (1 + creatinine [mg/dL]) + 0.94 × ln (1 + bilirubin [mg/dL]) + 1.658 × In (1 + international normalized ratio)^6^. UKELD score was calculated as 5.395 × In (international normalized ratio) + 1.485 × In (creatinine [μmol/L]) + 3.13 × In (bilirubin [μmol/L]) −81.565 × In (Na [mmol/L]) + 435^7^. The iMELD score was calculated as MELD + age (years) × 0.3–0.7 × Na (mmol/L) + 100 5. The MELD-Na score was calculated as MELD + (140 − Na [mmol/L]) − 0.025 × MELD × (140 − Na [mmol/L])^4^. The Na concentration is bound between 125 and 140 mmol/L. The MELD-AS score was calculated as MELD + 4.53 × (Na < 135 mmol/L [0.1]) + 4.46 × (persisted ascites [0.1])^3^. The conventional CTP score was calculated on the basis of serum bilirubin and albumin levels, the prothrombin time, as well as the presence and severity of ascites and encephalopathy. The modified CTP (mCTP) was obtained by assigning an additional 1 point to patients with serum bilirubin level >8 mg/dL, prothrombin time prolongation >11 sec, or albumin level <2.3 g/dL^10^.

### Statistical analysis

Quantitative variables were expressed as mean ± SD and categorical variables were presented as values and percentages. The area under the receiver operating characteristic curve (AUROC) was calculated for evaluating the predictive accuracy of prognostic scores at post-transplant mortality. A time-dependent AUROC was used for analyzing censored survival data^11^. AUROCs from various formulas were compared through a non-parametric approach that applied generalized U-statistics on the covariance matrix estimation with Hommel correction^12^. Kaplan-Meier method with log-rank test was used for cumulative survival comparison. Logistic regression analysis was used to evaluate risk of MELD on 3-month mortality. Pre-transplant variables were detected by univariate analysis and those with statistical significance were taken for a step-by-step multivariate regression analysis. According to the results of multivariate analysis, a new model for predicting 3-month mortality in patients with high pre-transplant prognostic score could be established. The method of Hosmer and Lemeshow was used to assess its goodness of fit. SAS software version 9.2 (www.sas.com, SAS institute, Cary, NC, USA) was used to complete all the analyses, and a *P* value of <0.05 was considered statistically significant^13^.

## Results

### Comparison between MELD and other seven formulas on predicting post-transplant mortality

The majority of study patients (n = 4,436/6,014) were diagnosed with HBV-related end-stage liver disease. As shown in Table 2, MELD, uMELD and MELD-AS had good predictive accuracies at 3-month (AUROC > 0.70). MELD presented the highest AUROCs at different time periods after transplantation, followed by uMELD, MELD-AS and MELD-Na, which represented almost similar predictive accuracies compared with MELD. By comparison of AUROCs, MELD had a significantly better prognostic predictive ability than iMELD, UKELD, CTP and mCTP (all *P* < 0.05).

There were (n = 509/6,014) patients diagnosed with HCV-related end-stage liver disease. MELD presented the highest AUROCs but UKELD showed the lowest ones at different time periods after transplantation. However, the differences between MELD and other formulas were not statically significant after Hommel correction (all *P* > 0.05).

There were (n = 314/6,014) patients receiving transplantation for alcohol-induced end-stage liver disease. uMELD and MELD-Na showed higher AUROCs than MELD at different time periods after transplantation, while mCTP represent the highest AUROCs at 6-month and 1-year. UKELD and CTP showed the lowest AUROCs. The comparison between AUROCs showed no significant difference after Hommel correction (all *P* > 0.05).

### The ability of MELD in predicting short-term post-transplant mortality

**Figure 1:**
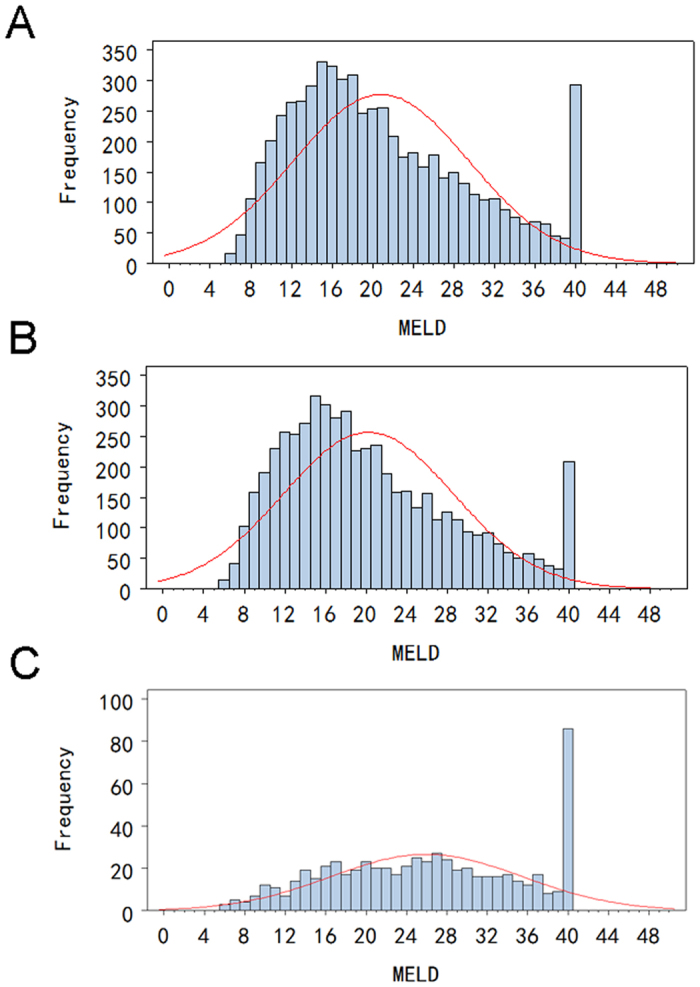
Distribution of MELD scores in cirrhotic patients undergoing liver transplantation: (**A**) all study patients; (**B**) patients survived at 3-month post-transplantation; (**C**) patients died within 3-month post-transplantation.

The distribution of MELD scores among all study patients were presented in Fig. 1A with a mean value of 20.8 ± 8.7. Mean MELD scores of patients died within 3 months after transplantation versus survivors were 25.3 ± 8.9 and 20.2 ± 8.4 (*P* < 0.001), respectively (Fig. 1B and C).

**Figure 2:**
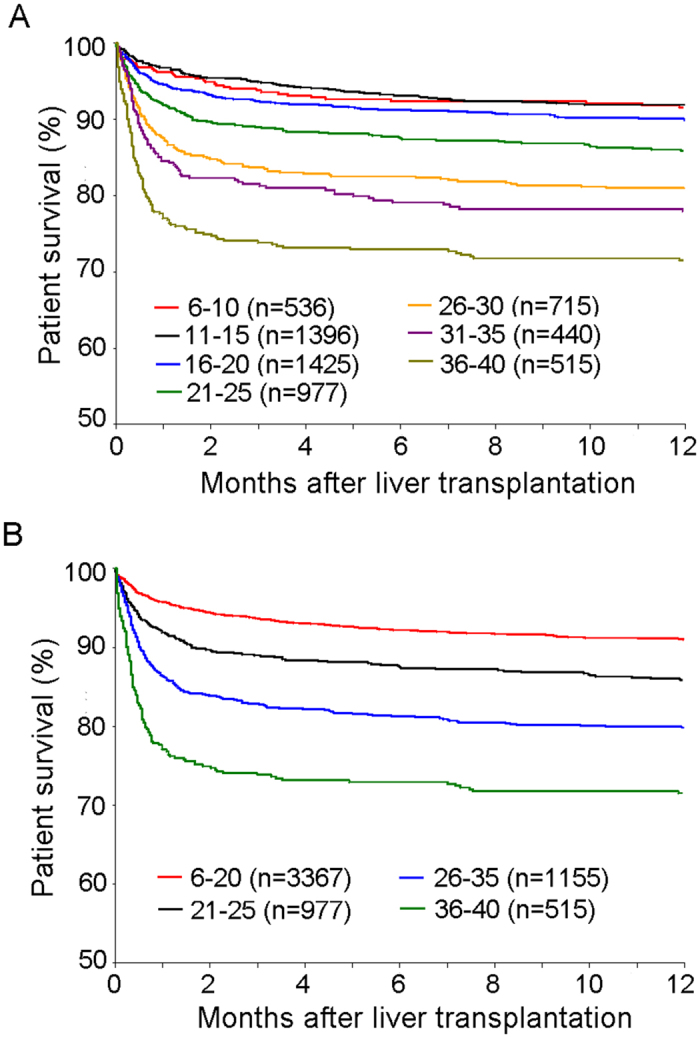
Comparison of short-term survival among patients with different MELD category: 6–10, 11–15, 16–20, 21–25, 26–30, 31–35 and 36–40 (**A**); 6–20, 21–25, 26–35 and 36–40 (**B**).

The study population was firstly stratified into seven groups according to their MELD scores: 6–10, 11–15, 16–20, 21–25, 26–30, 31–35 and 36–40. The Kaplan-Meier survival curve showed that patients of different MELD categories were associated with different post-transplant survivals (Fig. 2A). But the difference was not significant among three MELD < 21 groups (6–10, 11–15, 16–20), and between MELD 26–30 group and MELD 31–35 group (all *P* > 0.05). Therefore, we divided patients into four groups according to a new MELD score category: 6–20, 21–25, 26–35 and 36–40. Patient survivals in the four groups were of statistically significant difference (all *P* < 0.001) (Fig. 2B). The risk of poor survival increased significantly across the different MELD score categories (6–20, 21–25, 26–35 and 36–40). Patients with MELD score >35 showed the worst post-transplant survival, with a 3-month mortality rate of (26.2%, 167/515).

**Figure 3:**
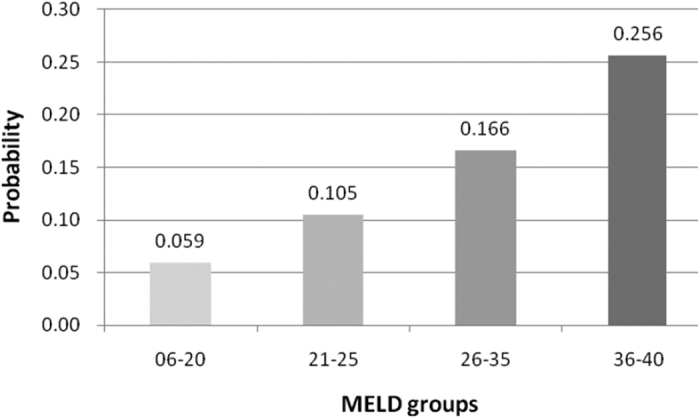
The probability of 3-month death according to different MELD category.

In addition, we also found that most of deaths (66.9%, 627/937) occurred during the first 3-month after transplantation. Thus, we took the 3-month death as the end point and found MELD score category (1: 6–20; 2: 21–25; 3: 26–35; 4: 36–40) was a risk factors with odds ratio 1.762 and 95% confidence internal 1.635–1.898 (*P* < 0.001). The probability of 3-month mortality increased sharply in higher MELD score categories (Fig. 3).

### Establishment of a model for identifying the sickest patients among those with MELD score >35

**Figure 4:**
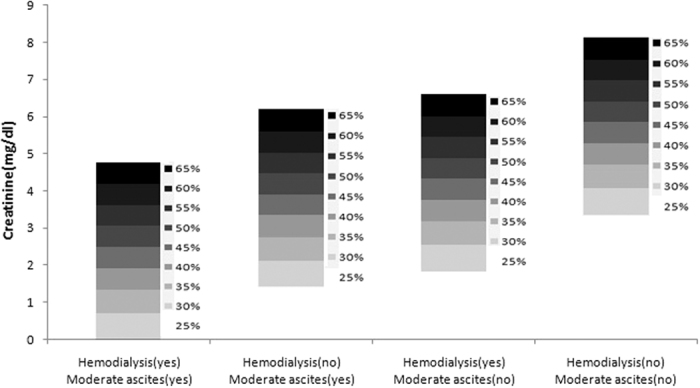
Probability of 3-month death (PD_3m_) following liver transplantation in patients with MELD score >35.

Among 515 patients with MELD > 35, 300 were selected for the construction of prognostic model and the other 215 were used as a validation group. Patients were selected according to the transplant time. Binary logistic regression analysis showed that serum creatinine, need for hemodialysis and moderate ascites were independent risk factors of 3-month mortality after transplantation (Table 3). A prognostic scoring was then established according to the multivariate analysis: risk score = −2.3090 + 0.3600 × Creatinine (mg/dl) + 0.5493 × (need for hemodialysis [0, 1]) + 0.7000 × (moderate ascites [0, 1]). Probability of Death at 3-month following liver transplantation (PD_3m_) = EXP (risk score)/[1 + EXP (risk score)] (Fig. 4). This model had a good fit (*P* = 0.521 to reject model fit) and predicted 3-month mortality after transplantation much better than MELD both in this group (AUC: 0.703 vs. 0.590, *P* = 0.023) and validation group (AUC: 0.737 vs. 0.589, *P* = 0.017). Patients in validation group with a high PD_3m_ score (>0.5) had a 3-month mortality rate of 66.7% (24/36).

## Discussion

Although there is a general consensus that MELD scoring system is an excellent predictor of patient mortality on the waiting list, it remains controversial that whether the pre-transplant MELD score could be a predictor of post-transplant survival as summarized by a systematic review^14^. In this large cohort national study, we found that MELD was a good predictor of short-term post-transplant mortality in different etiological groups, while UKELD and CTP were the worst predictors among eight prognostic scoring systems. Particularly in patients with HBV infection, MELD represented the best predictive ability while the incorporation of Na, age or ascites did not enhance its predictive ability. It looks like a inexplicable phenomenon because hyponatremia has been clearly shown to be an important predictor of both waiting list mortality^4^,^5^,^9^,^15^ and poor post-transplant outcomes^16^,^17^,^18^,^19^,^20^. One possible reason is that serum sodium concentration is variable in cirrhotic patients after therapeutic maneuvers such as water restriction or volume expansion, administration or withdrawal of diuretics, and intravenous hypotonic fluids, which may limit the prognostic value of serum sodium. Another possible reason is that in this study, serum sodium level was the last recorded value before transplantation, which may be corrected at least transiently by some measures such as the newly developed selective V2 receptor antagonist drugs^21^,^22^,^23^. It has been reported that a history of prior hyponatremia was associated with adverse post-transplant outcome, even if hyponatraemia was subsequently resolved^18^. Therefore, not a single value but rather a history of dynamic change of serum sodium concentration should be recorded to improve the prognostic accuracy of MELD incorporating Na formulas. In addition, pseudohyponatremia, the coexistence of hyponatremia and normal plasma tonicity, arises most commonly in situations of hyperlipidemia or hypergammaglobulinemia, which is not an uncommon phenomenon in patients with severe cirrhosis^24^. Pseudohyponatremia may not be associated with poor prognosis and this phenomenon should be paid attention in the differential diagnosis of real hyponatremia.

Because the performance of MELD showed superiority in the comparison of prognostic accuracy, we further verified its predictive ability by comparing cumulative survival in different MELD subgroups. Consistent with other studies^25^,^26^,^27^, the cut-off point of MELD score >35 was the suitable one for identifying patients with the highest risk of death after transplantation. Furthermore, we demonstrated that high serum creatinine level, renal failure requiring hemodialysis and moderate ascites led to dismal outcome in patients with MELD score >35. A new prognostic model based on these three parameters was then established and further verified in the validation group. This model represented good predictive accuracy and a threshold PD_3m_ 50% could clearly stratify patients into high-risk and low-risk groups. Patients in high-risk group were the sickest liver transplant candidates, who would probably die within 3 months after transplantation (the likelihood of death was more than one half). Considering that most liver transplantation centers have reported a 1-year survival rate of more than 85% in patients with benign underlying diseases, this model provides clinicians with a tool to identify patients who may not benefit from transplantation and therefore should be excluded.

This study has some limitations. First, because China is estimated to have the largest population of HBV world widely, HBV-related liver disease comprises the majority of liver transplantation causes in this study. The HCV and alcohol cohorts were small. Therefore, the result should be validated in some other studies, preferable to include other ethnic populations. Second, although D-MELD has been considered as a predictor of post-transplant survival, we did not evaluate its impact in our cohort due to data limitation.

In conclusion, MELD could be a valid predictor of short-term survival after liver transplantation compared with other seven formulas in patients with HBV-induced cirrhosis. Among patients with MELD score >35, a new prognostic model including serum creatinine, need for hemodialysis and moderate ascites can identify high-risk patients who should be excluded from waiting list to prevent wasteful transplantation.

## Additional Information

**How to cite this article:** Ling, Q. *et al*. Predicting short-term survival after liver transplantation on eight score systems: a national report from China Liver Transplant Registry. *Sci. Rep.* **7**, 42253; doi: 10.1038/srep42253 (2017).

**Publisher's note:** Springer Nature remains neutral with regard to jurisdictional claims in published maps and institutional affiliations.

## Figures and Tables

**Table 1:** Patient characteristics.

Characteristics	Values (n = 6,014)
Donor age (years)	29.8 ± 7.5
Donor male/female (n)	5,789/225
Donor sources (n)	
Living donor	506
Donation after cardiac death	5,508
Cold ischemia time (hours)	9.5 ± 7.8
Recipient age (years)	47.9 ± 9.7
Recipient male/female (n)	4,779/1,235
Abdominal operation history (n)	1,362
Etiology (n)	
HBV/HCV/alcohol/Other	4,436/509/314/755
Complications (n)	
Hepatic encephalopathy	1,517
Moderate ascites	4,273
Hepatorenal syndrome	411
Gastrointestinal bleeding	829
Spontaneous peritonitis	455
Blood group	
A/B/AB/O	1,891/1,712/604/1,807
Matched/Unmatched	5,911/103
Serum biochemistry	
Bilirubin (mg/dL)	10.5 ± 12.3
Creatinine (mg/dL)	1.0 ± 0.7
International normalized ratio	2.3 ± 3.7
Albumin (g/dL)	3.2 ± 0.6
Sodium (mmol/L)	138.8 ± 8.1

HBV, hepatitis B virus; HCV, hepatitis C virus.

**Table 2:** Comparison of AUROC to predict 3-month, 6-month and 1-year post-transplant mortality between MELD and other formulas in different etiologies.

	HBV (n = 4,436)		HCV (n = 509)		Alcohol (n = 314)	
	AUROC (95% CI)	*P* *	AUROC (95% CI)	*P* *	AUROC (95% CI)	*P* *
3- month						
MELD	0.704 (0.669–0.742)		0.665 (0.554–0.775)		0.635 (0.473–0.796)	
uMELD	0.704 (0.667–0.740)	0.729	0.659 (0.541–0.776)	0.718	0.646 (0.469–0.822)	0.912
MELD-Na	0.700 (0.663–0.737)	0.207	0.654 (0.539–0.770)	0.718	0.643 (0.504–0.783)	0.912
iMELD	0.679 (0.642–0.717)	0.004	0.649 (0.528–0.770)	0.718	0.629 (0.493–0.766)	0.912
UKELD	0.647 (0.606–0.687)	<0.001	0.607 (0.478–0.735)	0.516	0.610 (0.471–0.747)	0.912
MELD-AS	0.701 (0.665–0.738)	0.636	0.622 (0.501–0.744)	0.620	0.630 (0.480–0.779)	0.912
CTP	0.630 (0.592–0.668)	<0.001	0.621 (0.505–0.738)	0.718	0.599 (0.412–0.786)	0.912
mCTP	0.646 (0.608–0.684)	<0.001	0.607 (0.488–0.726)	0.718	0.622 (0.441–0.803)	0.912
6- month						
MELD	0.690 (0.655–0.726)		0.641 (0.532–0.751)		0.603 (0.441–0.792)	
uMELD	0.688 (0.653–0.724)	0.720	0.641 (0.527–0.756)	0.998	0.621 (0.449–0.792)	0.975
MELD-Na	0.685 (0.649–0.720)	0.210	0.627 (0.511–0.742)	0.998	0.611 (0.466–0.755)	0.975
iMELD	0.665 (0.630–0.701)	0.024	0.628 (0.512–0.745)	0.998	0.595 (0.454–0.738)	0.975
UKELD	0.638 (0.600–0.676)	<0.001	0.587 (0.463–0.710)	0.476	0.593 (0.460–0.726)	0.975
MELD-AS	0.686 (0.651–0.721)	0.646	0.600 (0.485–0.715)	0.636	0.602 (0.453–0.752)	0.975
CTP	0.622 (0.586–0.658)	<0.001	0.606 (0.499–0.714)	0.998	0.588 (0.412–0.764)	0.975
mCTP	0.639 (0.603–0.674)	<0.001	0.614 (0.506–0.722)	0.998	0.639 (0.467–0.810)	0.975
1- year						
MELD	0.679 (0.644–0.714)		0.640 (0.534–0.745)		0.590 (0.43645–0.7)	
uMELD	0.676 (0.641–0.711)	0.630	0.635 (0.525–0.745)	0.812	0.603 (0.438–0.767)	1.000
MELD-Na	0.675 (0.640–0.710)	0.519	0.623 (0.513–0.734)	0.804	0.593 (0.453–0.733)	1.000
iMELD	0.660 (0.625–0.695)	0.104	0.632 (0.523–0.732)	0.812	0.573 (0.433–0.713)	1.000
UKELD	0.631 (0.594–0.669)	<0.001	0.577 (0.460–0.693)	0.330	0.553 (0.409–0.698)	1.000
MELD-AS	0.677 (0.642–0.712)	0.630	0.593 (0.480–0.706)	0.330	0.591 (0.448–0.733)	1.000
CTP	0.611 (0.575–0.646)	<0.001	0.604 (0.502–0.706)	0.812	0.555 (0.378–0.731)	1.000
mCTP	0.627 (0.591–0.662)	<0.001	0.599 (0.491–0.707)	0.812	0.605 (0.432–0.777)	1.000

^*^: vs. MELD

Comparison of AUROCs was adjusted by Hommel correction. AUROC, area under the receiver operating characteristic curve; MELD, model for end-stage liver disease; HBV, hepatitis B virus; HCV, hepatitis C virus; UKELD, United Kingdom MELD; MELD-Na, MELD-sodium; iMELD, integrated MELD; CTP, Child-Turcotte-Pugh; uMELD, updated MELD; mCTP, modified Child-Turcotte-Pugh.

**Table 3:** Identification of risk factors of 3-month mortality in patients with MELD > 35.

Value	Univariate		Multivariate	
	Odds Ratio (95%CI)	*P*	Odds Ratio (95%CI)	*P*
Creatinine(mg/dl)	1.536 (1.304–1.809)	<0.001	1.433 (1.209–1.699)	<0.001
Hepatorenal syndrome	3.189 (1.898–5.357)	<0.001		
Need for hemodialysis	2.583 (1.540–4.333)	<0.001	1.732 (1.003–3.038)	0.048
Moderate ascites	2.530 (1.454–4.400)	0.001	2.014 (1.124–3.609)	0.019

MELD, model for end-stage liver disease; CI, confidence interval.
